# From passage to inhibition: Uncovering the structural and physiological inhibitory mechanisms of MCUb in mitochondrial calcium regulation

**DOI:** 10.1096/fj.202201080R

**Published:** 2022-12-20

**Authors:** Danielle M. Colussi, Peter B. Stathopulos

**Affiliations:** ^1^ Department of Physiology and Pharmacology, Schulich School of Medicine and Dentistry University of Western Ontario London Ontario Canada

**Keywords:** MCU, MCU dominant‐negative beta subunit, MCUb, mitochondrial calcium uniporter, mitochondrial calcium uptake, post‐translational modifications, structure–function

## Abstract

Mitochondrial calcium (Ca^2+^) regulation is critically implicated in the regulation of bioenergetics and cell fate. Ca^2+^, a universal signaling ion, passively diffuses into the mitochondrial intermembrane space (IMS) through voltage‐dependent anion channels (VDAC), where uptake into the matrix is tightly regulated across the inner mitochondrial membrane (IMM) by the mitochondrial Ca^2+^ uniporter complex (mtCU). In recent years, immense progress has been made in identifying and characterizing distinct structural and physiological mechanisms of mtCU component function. One of the main regulatory components of the Ca^2+^ selective mtCU channel is the mitochondrial Ca^2+^ uniporter dominant‐negative beta subunit (MCUb). The structural mechanisms underlying the inhibitory effect(s) exerted by MCUb are poorly understood, despite high homology to the main mitochondrial Ca^2+^ uniporter (MCU) channel‐forming subunits. In this review, we provide an overview of the structural differences between MCUb and MCU, believed to contribute to the inhibition of mitochondrial Ca^2+^ uptake. We highlight the possible structural rationale for the absent interaction between MCUb and the mitochondrial Ca^2+^ uptake 1 (MICU1) gatekeeping subunit and a potential widening of the pore upon integration of MCUb into the channel. We discuss physiological and pathophysiological information known about MCUb, underscoring implications in cardiac function and arrhythmia as a basis for future therapeutic discovery. Finally, we discuss potential post‐translational modifications on MCUb as another layer of important regulation.

**Abbreviations**
AD: Alzheimer's disease
ALL: acute lymphoblastic leukemia
AMPK: AMP‐activated protein kinase
ATP: adenosine 5′‐triphosphate
C^−^
: carboxyl
Ca^2+^
: calcium
CAMKII: calmodulin‐dependent protein kinase II
CCDC109B: coiled‐coil domain containing 109B protein
cryoEM: cryoelectron microscopy
DIME motif: Asp‐Ile‐Met‐Glu motif
DMD: Duchenne muscular dystrophy
EMRE: essential MCU regulator
ER: endoplasmic reticulum
GBM: glioblastoma multiforme
HA: hemagglutinin
HF: heart failure
HIF1α: hypoxia‐inducible factor‐1‐alpha
I/R: ischemia/reperfusion
IMM: inner mitochondrial membrane
IMS: intermembrane space
IP3R: inositol 1,4,5‐trisphosphate receptor
JML: juxtamembrane loop
KD: equilibrium dissociation constant
KO: knockout
Li^+^
: lithium
MAM: mitochondria‐associated membrane
MCU: mitochondrial calcium uniporter
MCUb: mitochondrial calcium uniporter dominant‐negative beta subunit
MCUB: MCUb gene
MCUR1: MCU regulator‐1
MICU1/2/3: mitochondrial calcium uptake 1, ‐2, and ‐3
mPTP: mitochondrial permeability transition pore
mROS: mitochondrial reactive oxygen species
mtCU: mitochondrial calcium uniporter complex
N^−^
: amino
Na^+^
: sodium
NCLX: sodium/calcium/lithium exchanger
OMM: outer mitochondrial membrane
PISA: protein, interfaces, structures, and assemblies server
PXP motif: Pro‐X‐Pro motif
Pyk2: proline‐rich tyrosine kinase 2
Ru360/265: Ruthenium red derivative 360 and ‐265
RyR2: ryanodine receptor 2
shRNA: short hairpin ribonucleic acid
siRNA: small interfering ribonucleic acid
SR: sarcoplasmic reticulum
STZ: streptozotocin
TM1/2: transmembrane domain 1 and ‐2
VDAC: voltage dependent anion channels
WT: wild‐type

## 1 INTRODUCTION

Mitochondria play essential roles in ATP production, cell death, and shaping cytosolic calcium (Ca^2+^) transients, with mitochondrial Ca^2+^ uptake into the matrix fundamentally regulating all of these processes.^1^, ^2^ Mitochondria have a large Ca^2+^ buffering capacity, taking up Ca^2+^ upon elevated cytosolic levels and protecting cells from Ca^2+^ overload.^3^, ^4^ Under physiological conditions, mitochondrial matrix Ca^2+^ plays a role in regulating key enzymes of the citric acid cycle and oxidative phosphorylation, such as pyruvate dehydrogenase, isocitrate dehydrogenase, α‐ketoglutarate dehydrogenase, and perhaps even the ATP synthase complex, to regulate cellular bioenergetics.^2^, ^5^, ^6^, ^7^ However, sustained excess matrix Ca^2+^ can be pathological due to mitochondrial permeability transition pore (mPTP) opening, the release of pro‐apoptotic factors, and a progressive signaling cascade leading to cell death.^8^, ^9^, ^10^

The endoplasmic reticulum (ER) and mitochondria are in close contact through mitochondria‐associated membranes (MAMs).^9^, ^11^, ^12^ Physiologic stimuli cause the ER to release large amounts of Ca^2+^ through inositol‐1,4,5‐trisphosphate receptor (IP_3_R) Ca^2+^ release channels, generating microdomains of elevated Ca^2+^ (i.e., >~10 μM) at the outer mitochondrial membrane (OMM).^13^ Subsequently, Ca^2+^ freely diffuses into the intermembrane space (IMS) through voltage‐dependent anion channels (VDAC).^10^, ^14^ Once in the IMS, Ca^2+^ uptake is tightly regulated across the inner mitochondrial membrane (IMM) by the mitochondrial Ca^2+^ uniporter complex (mtCU).^10^, ^15^ The driving force for Ca^2+^ uptake across the IMM through mtCU is the highly negative membrane potential (i.e., ~−180 mV).^1^, ^13^

After ~6 decades since the discovery of the mitochondrial Ca^2+^ uptake phenomenon by DeLuca, Engstrom, Vasington, and Murphy,^16^, ^17^, ^18^, ^19^ the identification of the molecular components making up mtCU began appearing in 2010.^20^ Several necessary components and accessory regulator proteins have been identified since that time (reviewed in Ref. [4, 21, 22]). Central to the expansion of the field included the electrophysiological characterization of mtCU by the Clapham group,^23^ and later the identification of the molecular components of mtCU, including the mitochondrial Ca^2+^ uptake‐1, 2 and 3 (MICU1/2/3) gatekeeping regulators by the Mootha group,^20^, ^24^ the mitochondrial Ca^2+^ uniporter (MCU) essential pore‐forming subunit by the Mootha and Rizzuto groups,^4^, ^25^, ^26^, ^27^ the essential MCU regulator (EMRE) by the Mootha group,^28^ the MCU regulator‐1 (MCUR1) scaffold factor by the Foskett and Madesh groups^29^ and the MCU dominant‐negative beta subunit (MCUb) by the Rizzuto group.^4^, ^30^ Remarkably, MCUb is a highly similar paralog of MCU, and research has shown the functional, physiologic, and pathophysiologic importance of MCU:MCUb expression ratios.^4^, ^30^ Nevertheless, there is currently a lack of understanding of the structural mechanisms underlying the MCUb inhibitory function despite high homology to the well‐studied MCU.

The most complete structural views of the human mtCU have been revealed by cryoelectron microscopy (cryoEM) and include MICU1, MICU2, MCU, and EMRE components.^31^, ^32^, ^33^ In this article, we provide an overview of the major mtCU components, regulators, and structural arrangements as revealed by the cryoEM structures and compare primary and higher‐order structures of MCU and homology‐modeled MCUb, exposing differences in protein–protein interactions and pore sizes in the context of mtCU. Our homology models use the first published mtCU cryoEM structures,^31^ reported by the Tsai and Feng groups. We review work characterizing MCUb in physiology and pathophysiology, led by the Rizzuto, Elrod, Belosludstev, Hajnóczky, and Mammucari groups, highlighting implications within cardiac function and arrhythmia, and survey potential post‐translational modifications within MCUb as an additional layer of regulation to this subunit.

## 2 MITOCHONDRIAL CALCIUM UNIPORTER COMPLEX (mtCU) COMPONENTS

**FIGURE 1:**
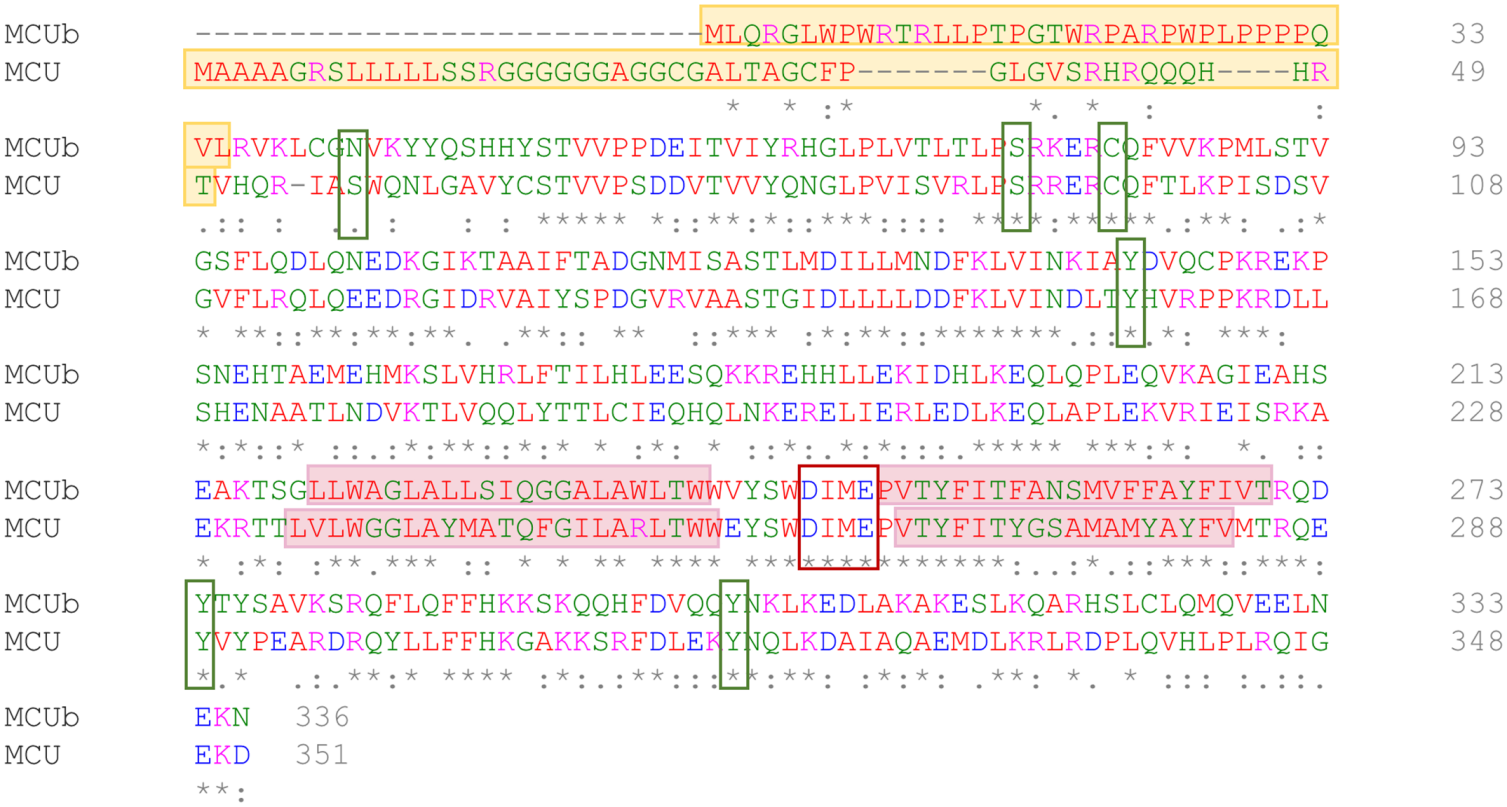
Human MCUb (NCBI accession # NP_060388.2) sequence alignment and conservation with human MCU (NCBI accession # NP_612366.1). Primary structure alignment of human MCUb and MCU conducted using Clustal Omega.^36^ The mitochondrial transit peptides are shaded yellow; the TM1 and TM2 domains are shaded pink, and the DIME motifs are outlined with a red box. Residues known to undergo post‐translational modifications in MCU and the corresponding residue in MCUb are highlighted with a green box. The residue numbers are shown at the right of the sequences. Below the alignment, (*) indicates a fully conserved residue; (:) indicates highly similar residues; (.) indicates conservation between residues with low similarity.

**FIGURE 2:**
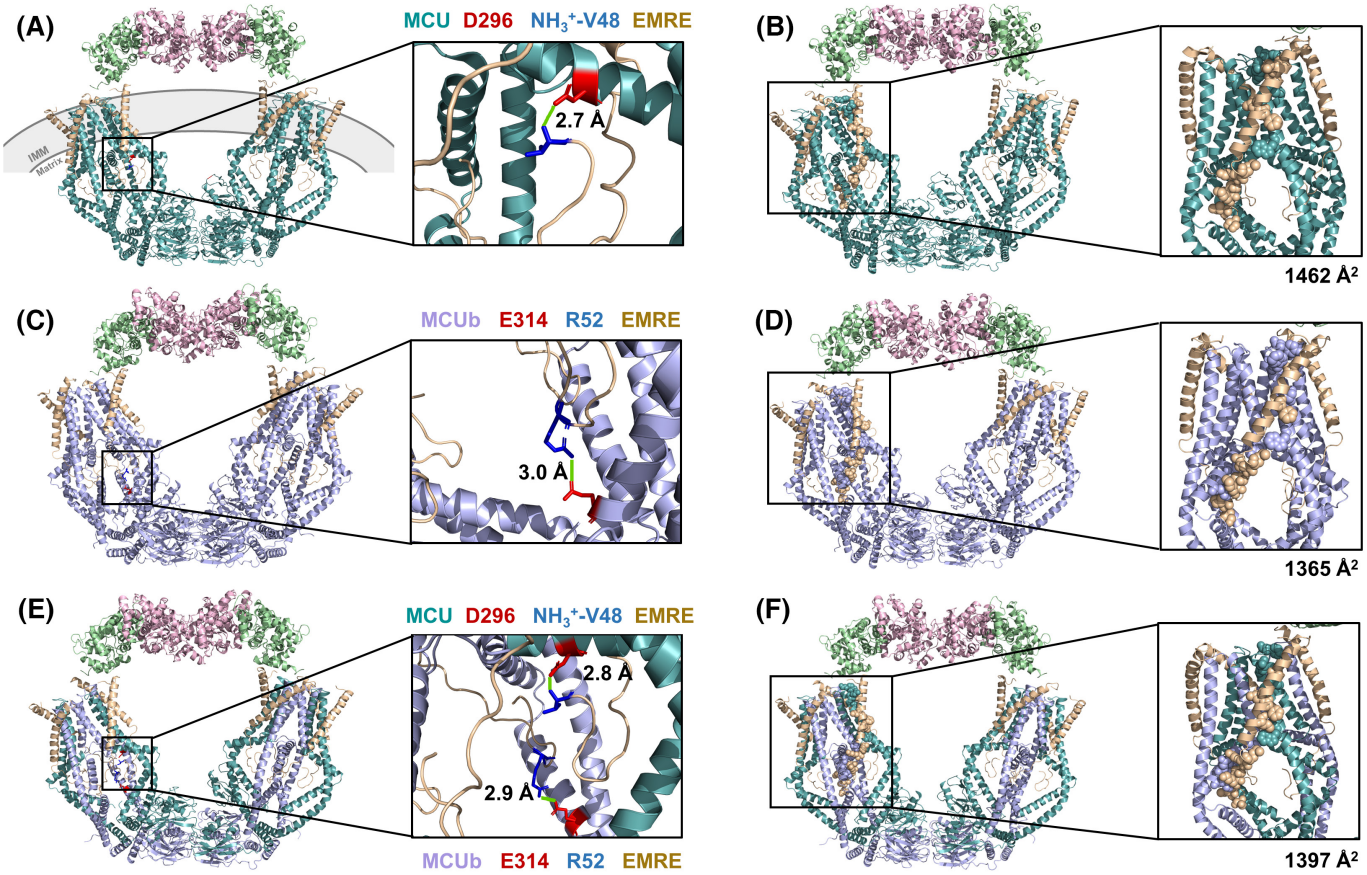
Three‐dimensional structures of human MCU (A) and MCUb (C) homotetrameric complexes and a 2 × MCU:2 × MCUb (E) heterotetrametric complex in high calcium (Ca^2+^). Each complex includes MCU (teal) or MCUb (lilac), EMRE (beige), MICU1 (green), and MICU2 (pink). At the right of (A), (C), and (E), a zoomed in view of the distinct salt bridges present in each respective complex model is shown with positive and negative charged residues as blue and red sticks, respectively. The closest distances between atoms involved in the salt bridge are indicated in each zoomed view (green line). Buried surface area exhibited by MCU (B) and MCUb (D) homotetrameric complexes and a 2 × MCU:2 × MCUb heterotetrameric complex (F), highlighting residues that are >50% buried in one interaction with EMRE (spacefill). At the right of (B), (D), and (F), a zoomed in view of a channel subunit interaction with EMRE is shown with the corresponding total buried surface area of the interaction indicated. The structure images were generated using the 6WDO.pdb and homology‐modeled coordinates in PyMOL. MCU, mitochondrial Ca^2+^ uniporter; MCUb, MCU dominant‐negative beta subunit; EMRE, essential MCU regulatory protein, MICU1/2, mitochondrial Ca^2+^ uptake 1 and −2 proteins; IMM, inner mitochondrial membrane.

MCU is the pore‐forming subunit that is essential for Ca^2+^ permeation into the matrix.^4^, ^26^, ^34^ MCU subunits preferentially oligomerize into active tetrameric channels, and these tetramers can dimerize along the curvature of the IMM to form larger V‐shaped complexes (see Section 3; Ref. [4, 12, 22, 31]). The MCU subunit consists of matrix‐oriented amino (N)‐ and carboxyl (C)‐ terminal domains with two coiled‐coil domains and two transmembrane domains (TM1 and TM2) that are linked by a short loop exposed to the IMS.^15^, ^35^ The critical Asp‐Ile‐Met‐Glu, DIME motif necessary for the Ca^2+^ selectivity of the channel is located at the end of the loop, forming the start of TM2 (Figure 1; Ref. [4, 22, 37]). The conserved DIME sequence^38^ of four MCU subunits is symmetrically arranged at the entrance of the oligomerized channel, creating an electronegative selectivity filter.^39^, ^40^ The Asp carboxylate ring of DIME formed at the entrance of the channel has a larger diameter compared to the Glu ring positioned just below, mediating Ca^2+^ coordination.^41^, ^42^ Conserved Trp and Pro residues located on each respective side of DIME may be essential for the orientation of the motif.^42^ This mtCU selectivity filter has a high Ca^2+^ affinity (equilibrium dissociation constant, *K*_*D*_, ≤2 nM) and a preference for binding Ca^2+^ over other cations.^23^, ^38^, ^39^, ^41^ The ion permeation pathway has two constriction points at opposing ends of the mtCU channel. The first is the Glu ring of DIME and the second is a luminal gate formed by a juxtamembrane loop (JML) at the exit of the transmembrane domain.^43^ To control the release of Ca^2+^ into the mitochondrial matrix, Glu288 and Val290 from the JML of each pore‐forming MCU subunit circle the pore exit to form the opening, with EMRE believed to promote the open conformation of this luminal gate.^33^, ^41^, ^43^, ^44^ EMRE interacts with three significant areas across two adjacent MCU subunits, including the side chain of Arg297 on MCU connecting the JML of MCU through a hydrogen bond to the backbone carbonyl of Val61 on EMRE.^43^ The large, matrix‐oriented N‐terminal domains of MCU self‐associate within and between channels (Figure 2; Ref. [31, 32, 33]), contain sites of regulatory post‐translational modifications (see Section 4) and bind cations to regulate assembly and channel activity.^1^, ^45^, ^46^

The MCU tetramer interacts with MICU1 directly or indirectly.^47^ MICU1 and MICU2 form a heterodimer with a gatekeeping function in mtCU channel activity.^37^, ^47^, ^48^ MICU3 is a paralog of the MICU proteins present in vertebrates and expressed at high levels in the central nervous system. MICU2 or MICU3 both form a disulfide bond‐mediated dimer only with MICU1 to enhance Ca^2+^ uptake.^49^ These gatekeeper proteins contain EF‐hand motifs that bind IMS Ca^2+^ and undergo conformational changes that may be involved in regulating the accessibility of Ca^2+^ to the mtCU channel pore or otherwise regulate channel open probability (see below; Ref. [31, 32, 33, 37, 50, 51, 52, 53]). Heritable loss of MICU1 and MICU2 function mutations leads to disorders characterized by muscle weakness, fatigue, lethargy, developmental delay, and learning disabilities.^21^, ^54^, ^55^, ^56^, ^57^ *MICU1* knockout (KO) mouse studies highlight a complex gatekeeping function as fibroblast KO mitochondria at low and high cytosolic Ca^2+^ show higher and lower rates of Ca^2+^ uptake, respectively,^58^ striated muscle KO mitochondria show increased basal but decreased Ca^2+^ uptake rates after stimulus^59^ and neuronal KO mitochondria show increased Ca^2+^ uptake at subthreshold cytosolic Ca^2+^ but reduced Ca^2+^ uptake at high cytosolic Ca^2+^.^60^

In low Ca^2+^, cryoEM revealed mtCU arrangements with MICU1 tightly occluding the pore entrance through electrostatic interactions between Lys/Arg residues of MICU1 and three of the DIME Asp261 residues of the MCU tetramer.^31^, ^32^ MICU2 does not contribute to the occlusion, primarily contacting MICU1 in this assembly. However, low Ca^2+^ mtCU arrangements have also been identified with MICU1 completely displaced from the pore, remaining part of the complex only via EMRE interactions. In this context, non‐occluded dimers of tetrameric channels, bridged through N‐terminal domain interactions of MCU in the matrix and MICU2 interactions in the IMS, have been resolved.^32^, ^33^ Non‐occluded, MICU2‐ and N‐terminal domain‐bridged tetrameric channels are also the dominant mtCU arrangement elucidated in high Ca^2+^.^31^, ^32^ The low Ca^2+^ occluded/high Ca^2+^ non‐occluded states provide a convenient explanation for MICU gatekeeping. Yet, this simple model is not only incompatible with the observed non‐blocked states in low Ca^2+^, but also compelling electrophysiology data collected in the absence of Ca^2+^ showing sodium (Na^+^) currents are similar in wild‐type (WT) and MICU1‐KO mitoplasts.^52^ At the same time, the Ca^2+^ currents in WT mitoplasts are ~2× that of MICU1‐KO mitoplasts due to MICU1‐dependent increases in the pore open probability.^52^ Given that EMRE bridges MICU1 and MCU in the human mtCU in high Ca^2+^ structures, we speculate that increased open probability occurs due to Ca^2+^‐dependent structural changes in the MICUs, which allosterically couple to the JML luminal gate of MCU via EMRE.^33^

Although not resolved in the mtCU cryoEM structures, the polyaspartate, IMS‐facing C‐terminus of EMRE interacts with a polybasic domain of MICU1, and the matrix‐oriented N‐terminus forms a beta‐hairpin motif, interacting with MCU.^37^, ^40^, ^43^, ^47^, ^61^ EMRE interacts with MCU at as high as a 1:1 ratio, and this EMRE:MCU interaction is necessary to create an active Ca^2+^ permeable channel in metazoans.^22^, ^44^ However, a 4 × EMRE:4 × MCU ratio is not required for channel functionality, with channel complexes containing 1–4 EMRE subunits having partial to full gatekeeping function.^62^ Concatemers enforcing 2 × EMRE:4 × MCU subunits recapitulated the activity, gatekeeping, and size of endogenous channels.^62^ MCUR1 is another regulatory subunit of mtCU, binding to MCU and EMRE.^22^, ^63^ MCUR1 acts as a scaffolding protein for the oligomerization of mtCU^22^, ^63^; however, MCUR1 may have other important roles in the regulation of the mPTP and/or as a complex IV assembly factor.^29^, ^64^, ^65^

Finally, MCUb, the dominant‐negative beta subunit, is a paralog of the MCU pore‐forming subunit.^66^ MCUb was initially introduced to the field as the coiled‐coil domain‐containing 109B (CCDC109B) protein.^22^, ^30^ In humans, MCUb shares 48.8% sequence identity and 83.9% similarity with MCU (Figure 1). Despite this high similarity, hetero‐oligomerization with MCU remarkably exerts an inhibitory effect on Ca^2+^ permeation through the mtCU channel.^66^ We explore potential structural, physiological, and pathophysiological mechanisms of MCUb‐mediated inhibition of the mtCU channel in subsequent sections.

## 3 MCU DOMINANT‐NEGATIVE BETA SUBUNIT (MCUb)

### 3.1 MCUb protein structure

MCUb consists of two coiled‐coil domains and two TM domains that are separated by a short IMS loop.^27^, ^30^ Despite the ~49% sequence identity between MCUb and MCU, one key amino acid differs between MCU and MCUb within the short loop.^27^, ^30^, ^37^ In MCU, Glu257 is thought to be critical for Ca^2+^ permeation through the mtCU channel.^30^, ^37^ The Glu257 in MCU is replaced by Val242 in MCUb, resulting in the loss of a negative charge.^30^, ^67^ Thus, the surface of MCUb is less electronegative than MCU, which may interfere with electrostatic attraction and Ca^2+^ permeation through the mtCU channel.^30^, ^67^

Proteomic data show that, unlike MCU, MCUb does not interact with MICU1 through EMRE.^37^ Immunoprecipitation experiments performed with *MCU* ^*−/−*^ KO HeLa cells revealed that EMRE fused with a C‐terminal FLAG tag interacts with both MCU and MCUb fused to C‐terminal HA tags.^66^ Further, MCUb‐HA was confirmed to interact with MCU‐FLAG but not MICU1‐FLAG or MICU2‐FLAG by pull‐down.^66^ Here, we used the high Ca^2+^/non‐occluded (6WDO.pdb; Ref. [31]) and low Ca^2+^/occluded (6WDN.pdb; Ref. [31]) human MCU‐EMRE‐MICU1‐MICU2 structures, to model the consequences of human MCUb incorporation into the mtCU assembly. The complex structures are composed of 4 × MCU:4 × EMRE:1 × MICU1:1 × MICU2 subunits per channel. The homology modeling was performed using Modeler^68^ and the human MCUb sequence (NCBI accession # NP_060388.2), and the protein–protein interaction analysis was done using the protein, interfaces, structures, and assemblies (PISA) server.^69^ We created 2 × MCU:2 × MCUb heterocomplexes with alternating MCU/MCUb proteins (i.e., MCU:MCUb:MCU:MCUb) and MCUb homotetramers for analysis. Note that symmetry restraints were not enforced, so analogous interfaces between the same subunit types were not always identical.

In comparison to the 6WDO.pdb non‐occluded template structure in high Ca^2+^ (Figure 2A,B), the high Ca^2+^ MCUb homotetramer complex strikingly revealed the loss of a salt bridge between the N‐terminus of EMRE and the channel forming subunit (Figure 2C,D). In the template mtCU channel, a salt bridge exists between Asp296 of MCU and the N‐terminus (i.e., NH_3_^+^) at Val48 of EMRE. Note that residues 1–47 of EMRE comprise the mitochondrial transit sequence of the resolved model, while UniProtKB accession NX_Q9H4I9 reports the mitochondrial transit sequence of human EMRE as residues 1–52. In contrast, while a salt bridge was predicted between Glu314 on MCUb and Arg52 on EMRE (Figure 2C), the location of this charge interaction neither involves the EMRE N‐terminus nor the position on MCUb that aligns with MCU Asp296. Asp296 is not conserved in MCUb, where it exists as Ser281 (Figure 1). Arg252 of MCU is thought to be critical for Ca^2+^ permeation and exists as Trp237 in MCUb.^27^, ^30^, ^37^ PISA identified hydrogen bonds between Arg252 of MCU and Thr82 as well as Ser85 of EMRE, with the Arg252:Thr82 hydrogen bond lost upon MCUb integration (Table 1). Notably, the buried surface area was smaller for the MCUb‐EMRE interaction compared to MCU‐EMRE (Table 1). Interestingly, our high Ca^2+^/non‐occluded MCU‐MCUb heterotetramer complex model (Figure 2E,F) showed a decrease in the number of intermolecular hydrogen bonds between MCU and MCUb and an increase in the number of intermolecular salt bridges between MCU/MCUb and EMRE compared to either the MCU or MCUb homotetramer structures (Table 1). Further, the buried surface area between the MCUb‐MCUb interfaces was greater than the MCU‐MCUb or MCU‐MCU interfaces (Table 1). In addition, the MCUb‐MCUb interactions showed the highest number of distinct hydrogen bonds and salt bridges among all channel‐forming subunit interactions (Table 1).

**TABLE 1:** Summary of interface properties for the MCU homotetramer, MCUb homotetramer, and MCU‐MCUb heterotetramer complexes in high Ca^2+^/non‐occluded and low Ca^2+^/occluded states

Interface composition interactions	Number of interface atoms	Number of interface residues	Number of surface atoms	Number of surface residues	Buried Surface Area (Å^2^)^c^	Number of distinct hydrogen bonds^b^	Number of distinct salt bridges^b^
*High Ca* ^*2+*^ */non‐occluded models* ^d^							
MCU homotetramer							
MCU‐MCU	2072	532	17 877	3171	10 065	34	4
MCU‐EMRE	1206	323	14 502	2524	5926	13	1
MCU‐MICU1	20	4	2856	528	69	0	0
EMRE‐MICU1	74	23	3372	625	293	0	1
MCUb homotetramer							
MCUb‐MCUb	2362	612	18 768	3258	10 841	39	7
MCUb‐EMRE	1169	318	15 082	2582	5596	13	1
MCUb‐MICU1	15	4	3063	555	63	0	0
EMRE‐MICU1	81	24	3663	671	382	1	1
MCU‐MCUb heterotetramer^a^							
MCU/MCUb	2046	535	18 138	3213	9834	32	6
MCU/MCUb‐EMRE	1149	311	14 658	2552	5678	16	2
MCU/MCUb‐MICU1	94	7	2882	532	94	0	0
EMRE‐MICU1	75	23	3287	621	365	3	3
*Low Ca* ^*2+*^ */occluded models* ^e^							
MCU homotetramer							
MCU‐EMRE	1121	300	10 686	1748	5551	10	1
MCU‐MICU1	269	71	10 718	2016	1109	8	3
EMRE‐MICU1	58	17	3910	765	251	0	0
MCUb homotetramer							
MCUb‐EMRE	1068	293	10 646	1744	5214	10	1
MCUb‐MICU1	269	72	10 807	2034	1090	8	3
EMRE‐MICU1	57	17	3944	775	252	1	0
MCU‐MCUb heterotetramer^a^							
MCU/MCUb‐EMRE	1087	306	10 646	1746	5304	13	1
MCU/MCUb‐MICU1	248	66	10 723	2047	1042	10	4
EMRE‐MICU1	59	16	3925	781	249	1	0

^a^ The MCU‐MCUb heterotetramer models were generated with a 2 × MCU:2 × MCUb ratio of MCU to MCUb subunits within each channel tetramer.

^b^ Only unique hydrogen bonds and salt bridges are indicated.

^c^ Figure 2 highlights a single chain interaction between EMRE and MCU or MCUb in the high Ca^2+^/non‐occluded models; here, totals of all interacting chains are given.

^d^ The 6WDO high Ca^2+^/non‐occluded model consists of two dimerized mtCU channels.

^e^ The 6WDN low Ca^2+^/occluded model consists of one mtCU channel; MCU‐MCU/MCUb interactions are not given because the N‐terminal domains involved in these interactions are not resolved.

We also generated homology models using the occluded/low Ca^2+^ MCU‐EMRE‐MICU1‐MICU2 structure (6WDN.pdb; Ref. [31]) but since the N‐terminal domains of MCU are not resolved, MCU‐MCU, MCUb‐MCUb or MCU‐MCUb interactions, which involve the N‐terminal domains, were not compared. Nevertheless, PISA reported that the low Ca^2+^/occluded model showed less MCU‐EMRE total buried surface area compared to the high Ca^2+^/non‐occluded. Integration of MCUb either as a homotetramer or 2 × MCU:2 × MCUb heterocomplex resulted in less MCUb/MCU‐EMRE total buried surface area, similar to observations with non‐occluded models (Table 1). As expected, the low Ca^2+^/occluded structure shows ~16× more buried surface area between MCU‐MICU1 compared to non‐occluded, and integration of MCUb marginally decreased this buried surface area with MICU1 (Table 1). Collectively, our homology models suggest a smaller interface between MCUb and EMRE, highlighted by decreased buried surface area and altered salt‐bridge formation. Further, while the MCU‐MCUb interface showed less hydrogen bonds, the MCUb‐MCUb interface showed a larger buried surface area, number of hydrogen bonds, and salt bridges compared to the MCU homotetramer (Table 1). The weakened/smaller interface between MCUb and EMRE, shown in both occluded and non‐occluded models, may play a role in the inhibitory mechanism of MCUb, while the enhanced MCUb‐MCUb solvent‐inaccessible interface may promote MCUb‐MCUb assembly. The N‐terminal domain of EMRE contains an extended linker region with a highly conserved PXP motif, thought to stabilize the luminal gate of MCU in an open conformation due to the rigidity of Pro.^41^, ^44^ Thus, a tighter MCU interaction (expanded interface) with EMRE could promote the opening of the mtCU luminal gate for Ca^2+^ uptake into the matrix, while a weaker MCUb‐EMRE interaction could suppress open probability.

**FIGURE 3:**
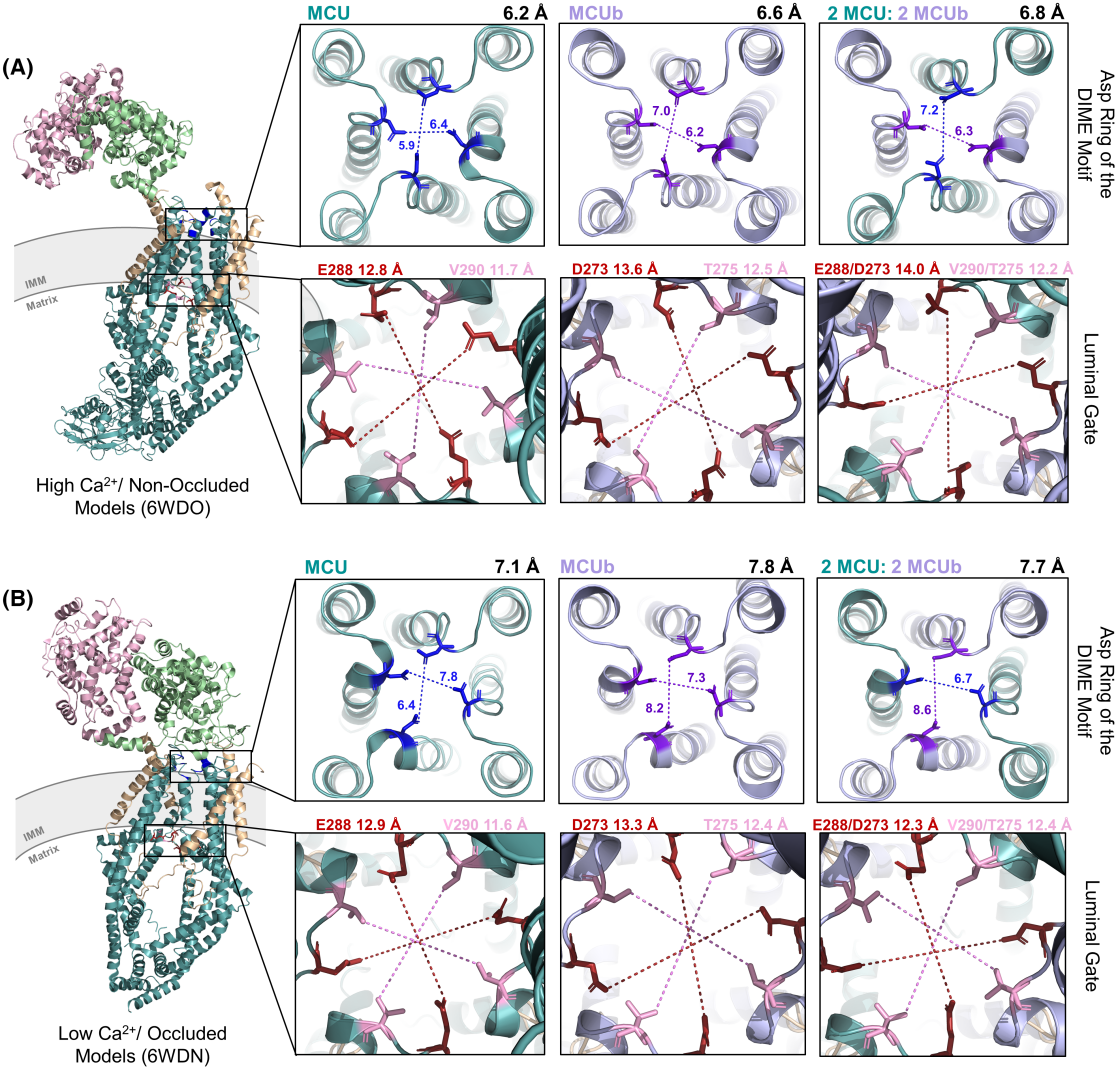
mtCU pore entry and exit diameters of the Ca^2+^ selectivity filter and the luminal gate of human MCU homotetrameric, MCUb homotetrameric, and 2 × MCU:2 × MCUb heterotetrameric complex structures elucidated in (A) high Ca^2+^/non‐occluded and (B) low Ca^2+^/occluded states. mtCU complexes include MCU (teal) or MCUb (lilac), EMRE (beige), MICU1 (green), and MICU2 (pink). The shortest distances are indicated between oxygens of MCU Asp261 (blue) and MCUb Asp246 (purple) from the DIME motifs of opposing subunits in each direction. The average of the shortest distances measured crosswise is indicated at the top right of each box in the first and third rows. The shortest distances were measured between opposing Glu288/Asp273 (brown) or Val290/Thr275 (pink) residues, the two residues that form the luminal gate in MCU/MCUb, respectively, with the average of these distances indicated at the top of each box in the second and fourth row. The structure images were generated using 6WDO.pdb and 6WDN.pdb for non‐occluded and occluded states, respectively, and homology‐modeled coordinates in PyMOL. MCU, mitochondrial Ca^2+^ uniporter; MCUb, MCU dominant‐negative beta subunit; IMM, inner mitochondrial membrane.

Previous homology modeling of human MCU, human MCUb, and MCU‐MCUb heteromeric channels based on the *Aspergillus fischeri* (fungal) MCU structure, suggested that there are fewer hydrophobic interactions between TM1 and TM2 from the adjacent MCU subunit of the complex, resulting in a widening of the IMS side of the pore.^37^, ^67^ The decreased hydrophobic interactions are due to the replacement of hydrophobic side chains in MCU with polar/small neutral in MCUb (i.e., specifically Ala244 and Phe247 exist as Ser229 and Gly232 in MCUb; Figure 1). Indeed, the reported diameter of the pore measured in fungal structure‐derived human MCU/MCUb models changed from 7.0 Å in the human MCU homotetramer to 10.4 Å in the MCUb homotetramer to 8.9 Å in the human heterotetramer with 3 × MCU:1 × MCUb subunits (6D7W.pdb; Ref. [37]). Two constriction points at opposite ends of the Ca^2+^ permeation pathway through mtCU are the Asp and Glu rings of DIME at the IMS pore entrance and the luminal gate at the matrix pore exit. In our models based on resolved human mtCU structures, the Glu ring of DIME did not exhibit any notable diameter changes after MCUb integration in either the occluded or non‐occluded states, in contrast to the DIME Asp ring distances, which changed. Specifically, The high Ca^2+^/non‐occluded human mtCU template structure showed an average shortest pore diameter of 6.2 Å, measured between Asp261 of opposite MCU subunits. This distance increased to 6.6 Å upon integration of MCUb, modeled with 2 × MCU:2 × MCUb, and further increased to 6.8 Å in the MCUb homotetramer (Figure 3A). Further, the low Ca^2+^/occluded mtCU template structure showed an average shortest pore diameter of 7.1 Å between Asp261 residues, which increased to 7.8 Å for the MCUb homotetrameric complex and 7.7 Å in the 2 × MCU:2 × MCUb heterotetrameric complex (Figure 3B).

The second constriction point includes Glu288 and Val290 of MCU, making up the luminal gate; these residues are not conserved in MCUb, existing as Asp273 and Thr275 (Figure 1). The shortest pore distances between these JML‐forming residues of the luminal gate increased upon the incorporation of MCUb into mtCU. In the high Ca^2+^/non‐occluded human mtCU template structure, the Glu288 and Val290 average shortest pore diameter increased from 12.8 and 11.7 Å in the MCU homotetrameric complex, respectively, to 13.6 and 12.5 Å determined for the Asp273 and Thr275 residues in the MCUb homotetrameric complex, respectively (Figure 3A). The diameter was also increased in the 2 × MCU:2 × MCUb heterotetrameric complex (i.e., to 14.0 and 12.2 Å, respectively). In the low Ca^2+^/occluded mtCU template structure, the Glu288 and Val290 average shortest pore diameter increased from 12.9 and 11.6 Å in the MCU homotetrameric complex, respectively, to 13.3 and 12.4 Å determined for the Asp273 and Thr275 residues in the MCUb homotetrameric complex, respectively (Figure 3B). The diameter was also increased in the 2 × MCU:2 × MCUb heterotetrameric complex for the Val290/Thr275 pairs only (i.e., to 12.4 Å). Thus, consistent with previous models based on a fungal MCU structure,^37^ integration of MCUb increases distances between DIME Asp residues, as well as between JML residues of the luminal gate, which could perturb Ca^2+^ attraction, coordination, gating, and permeation^70^ and/or MICU1 and EMRE interactions.

MCUb alone does not form a Ca^2+^ permeable channel when inserted into lipid bilayers.^30^, ^67^ Nevertheless, MCUb does form a channel permeable to Na^+^ ions.^30^, ^37^ Therefore, many questions remain about the precise residue differences in the MCUb structure that result in the inhibitory effects of the subunit.^37^ Similarly, the mechanisms underlying precisely how the MCUb subunits integrate into a channel remain unclear. Elucidation of the high‐resolution structure of MCUb in the context of an assembled homomeric and heteromeric channel will help pinpoint precise residues underlying inhibition and differences in complex assembly; further, this structural data will guide future research aimed at answering elusive questions surrounding the impact of post‐translational modifications and disease‐related mutations on the structure and function of MCUb.

### 3.2 MCUb protein function

**FIGURE 4:**
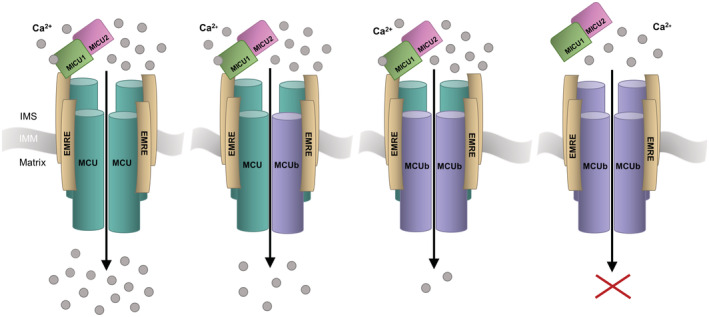
Schematic of mitochondrial Ca^2+^ uniporter (MCU) complexes with varying MCU:MCUb subunit ratios and the respective Ca^2+^ permeation. With an increasing expression of MCUb displacing MCU from the tetrameric channel, Ca^2+^ uptake into the matrix decreases. The MCUb homotetrameric complex does not form a Ca^+^ permeable channel, and the MICU gatekeeping proteins may not interact. MCUb, MCU dominant‐negative beta subunit; EMRE, essential MCU regulatory protein, MICU1/2, mitochondrial Ca^2+^ uptake 1 and −2 proteins; IMS, intermembrane space; IMM, inner mitochondrial membrane. Adapted from Ref. [4].

MCUb, a paralog of MCU, acts as a dominant negative inhibitor of the mtCU channel.^30^ MCUb hetero‐oligomerizes with MCU, integrating into mtCU, to negatively regulate Ca^2+^ uptake into the mitochondrial matrix.^9^, ^30^ Initial findings suggested that MCUb must integrate into the complex during assembly rather than a pre‐existing mtCU channel.^30^ Co‐expression of MCU and MCUb resulted in a dramatic decrease in mitochondrial Ca^2+^ uptake compared to complexes made up of MCU alone, supporting the inhibitory role of MCUb when incorporated into mtCU.^30^ More recent work has shown that MCUb can integrate into functional channels, with MCUb expression displacing MCU channel subunits from mtCU and decreasing the association with MICU1.^66^ This decreased association between mtCU and MICU1 may be due to perturbed interactions between MCUb and EMRE (see above; Ref. [37]). When MICU1 binds Ca^2+^, mtCU open probability increases, boosting Ca^2+^ permeation when cellular energy demand rises.^52^ Thus, higher MCUb:MCU stoichiometric ratios suppress Ca^2+^ uptake into the matrix (Figure 4; Ref. [66, 71]).

*MCUB* deletion affects mtCU assembly and functional capacity.^66^, ^71^ Deletion of *MCUB* using CRISPR/Cas9n resulted in a ~ 2.7‐fold and ~ 4.5‐fold increased expression of MCU and EMRE, respectively, compared to WT HeLa cells.^66^ The loss of *MCUB* increased the mitochondrial Ca^2+^ uptake amplitudes by ~80%.^66^ The increased Ca^2+^ uptake function decreased cytosolic Ca^2+^ peaks following the release of Ca^2+^ from the ER.^66^, ^71^ Thus, *MCUB* KO cells are readily susceptible to mitochondrial Ca^2+^ overload and mPTP opening.^66^, ^71^ Indeed, the *MCUB* KO cells withstood ~30% less bath Ca^+^ load in a permeabilized cell system before the mitochondrial membrane potential was lost.^66^

Currently, there are no effective therapies to prevent or reduce mitochondrial Ca^2+^ overload in disease.^37^ Ruthenium Red derivatives, such as Ru360, are the best current pharmacological approaches available to block mitochondrial Ca^2+^ uptake.^37^ They directly bind to the solvent‐exposed Asp‐ring of the DIME selectivity filter of MCU, inhibiting channel function.^37^, ^72^ Ru360 lacks specificity and is unable to pass cell membranes easily.^30^ More recent Ruthenium Red derivatives such as Ru265 show improved cell permeability and the potential to inhibit the channel by binding to regions outside the pore.^35^ Nevertheless, Ruthenium‐based compounds are not used in clinical settings. It is interesting to speculate that MCUb could represent an alternative approach to inhibiting mtCU‐mediated Ca^2+^ uptake and mPTP opening.^30^ For example, compounds that stabilize MCUb or mechanisms inducing increased MCUb expression could promote inhibition of the mtCU channel.

### 3.3 The role of MCUb in human physiology

The *MCUB* gene is located on chromosome 4 in *Homo sapiens*.^30^ The translated ~340 amino acid (~39 kDa) protein^30^ is highly conserved among vertebrates but not found in other organisms containing MCU, such as plants, kinetoplastids, nematodes, and arthropods.^30^ MCUb is differentially expressed compared to MCU, with high variability between tissue types, perhaps due to the distinct mitochondrial Ca^2+^ uptake demands across tissues.^30^, ^71^ The ratio of MCU:MCUb expressed can vary from around 3:1 within the heart or lung to greater than 40:1 within skeletal muscle.^30^, ^38^, ^40^ Analyses of the regulation of both MCU and MCUb under specific conditions, such as during development or pathological states, would likely reveal instances where the MCU:MCUb ratio is altered, in line with the metabolic Ca^2+^ demand.^30^ Well‐regulated mitochondrial Ca^2+^ uptake contributes to the homeostasis of many organs involved in systemic metabolisms, such as the liver, skeletal muscle, adipose tissue, and heart.^9^

In the normal physiologic heart, there is low MCUb expression and thus, MICU1 remains coupled to the channel with a low MICU1:MCU protein abundance ratio to enhance open probability.^71^, ^73^ Thus, this type of complex assembly results in a reduced threshold for Ca^2+^ uptake, allowing mitochondrial Ca^2+^ oscillations to occur on a beat‐by‐beat basis.^71^, ^74^ The potentially increased baseline mtCU channel activity within cardiac mitochondria could lead to increased metabolic flexibility, with the ability to take up more Ca^2+^ into the matrix, but inversely cause cardiac mitochondria to become more sensitive to Ca^2+^ overload with a higher probability of the mPTP opening.^30^, ^75^

### 3.4 MCUb‐associated pathophysiologies

There have been no heritable mutations identified in MCUb linked to human disease.^67^ However, mitochondrial Ca^2+^ signaling dysregulation and alterations in MCUb expression have been observed as secondary characteristics of many human pathologies, ranging from musculoskeletal disease, neurodegenerative disease, cancer, and diabetes to heart disease.

In Duchenne muscular dystrophy (DMD), aberrant mitochondrial Ca^2+^ regulation results in impaired bioenergetics and progressive degradation of the skeletal muscle.^76^, ^77^ Dubinin et al. (2020b) determined that the quantity of MCUb protein in the skeletal muscle of DMD‐model mice was ~1.4‐fold greater than WT mice.^76^ Additionally, the MCU:MCUb ratio decreased by ~1.7‐fold in the diseased mitochondria.^76^ Thus, the reduced Ca^2+^ uptake observed in the DMD mitochondria may be associated with increased MCUb expression.^76^

Alzheimer's disease (AD) has also been associated with neuronal mitochondrial Ca^2+^ signaling impairment.^78^ Frontal cortex tissue from non‐familial, sporadic AD patients showed a considerable decrease in mitochondrial Na^+^/Ca^2+^/lithium (Li^+^) exchanger (NCLX) expression, the primary mitochondrial Ca^2+^ efflux mediator in excitable cells, along with a decrease in MICU1 and MCUb.^78^ These expression profile changes may contribute to the matrix Ca^2+^ overload reported in neurons during disease progression.^78^ Elevated mitochondrial Ca^2+^ levels preceded neuronal death and thus, may be a target for the development of neuroprotective therapies for AD.^79^

MtCU function has been highly implicated in cancer. The disturbance of Ca^2+^ homeostasis within cancer cells has been correlated with continuous cell proliferation and inhibition of cell death.^80^ Aberrant expression of MCUb has been associated with the malignant properties of gliomas.^81^ Consistent with this finding, the Cosmic Cancer Catalogue has identified 94 somatic mutations across the *MCUB* gene.^82^ In fact, Xu et al. (2017) determined that MCUb is a prognostic marker in glioma patients.^81^ Specifically, MCUb was highly expressed in high‐grade gliomas compared to almost no expression in normal brain tissue; further, high MCUb expression correlated with an increased tumor grade.^81^ Silencing of *MCUB* using shRNAs in U87MG and U251 human glioma cell lines inhibited proliferation, migration, and invasion and led to decreased tumor volume and prolonged overall survival in orthotopic tumor models of nude mice.^81^, ^83^ Glioblastoma multiforme (GBM) samples showed high expression of MCUb and hypoxia‐inducible factor 1‐α (HIF1α), a transcriptional regulator typically induced by hypoxia, both localized in areas bordering necrosis.^81^ Culturing U87MG and U251 cells under hypoxia resulted in ~2‐fold increase in *MCUB* mRNA levels, and further treatment with siRNAs targeting *HIF1α* reduced mRNA levels of *MCUB*.^81^ Thus, MCUb may be a potential tumor promotor in glioma progression, which is drastically upregulated under hypoxia and could be used as a prognostic marker or targeted as a novel treatment in human glioma.^81^

MCUb overexpression has been reported in drug‐resistant acute lymphoblastic leukemia (ALL).^84^, ^85^ Additionally, Tosatto et al. (2016) demonstrated correlations between MCU and MCUb expression levels and clinically presented breast cancer stages.^86^ Specifically, MCU expression was found to increase with tumor progression, while MCUb expression decreased.^86^ A positive correlation between *MCU* expression and *HIF1α* was shown in triple‐negative breast cancer samples.^86^ Thus, enhanced mitochondrial Ca^2+^ uptake due to increased MCU and decreased MCUb expression may promote HIF1α signaling, leading to increased tumor size and lymph node infiltration in breast cancer.^86^ Collectively, MCUb may contribute to cancer progression through the promotion of cell proliferation and migration and may reveal a therapeutic target for clinical intervention in multiple aggressive cancers.^81^, ^86^

Diabetes mellitus is also associated with mitochondrial Ca^2+^ dysfunction.^87^ Belosludtsev et al. (2019) found the rate of mitochondrial Ca^2+^ uptake increased ~1.4‐fold in hepatic cells from type 1 diabetic rats, 2 weeks after diabetes induction using streptozotocin (STZ).^87^ The levels of MCU and MICU1 expression did not change; however, the MCUb protein decreased by ~2‐fold.^87^ Thus, the MCU:MCUb ratio increased from ~3.0 in control rats to ~6.7 in diabetic rats.^87^ Therefore, the increased mitochondrial Ca^2+^ uptake within liver mitochondria of diabetic rats may be related to the decrease in MCUb expression.^87^

Finally, MCUb expression may be an endogenous cardioprotective mechanism, reducing mitochondrial Ca^2+^ overload‐induced injury.^88^ Despite being undetected in the adult mouse heart under normal conditions, MCUb expression was found to increase following ischemic injury.^66^, ^75^, ^88^ The increase in MCUb protein levels was observed only after 3 days post‐ischemia/reperfusion (I/R) in the hearts of mice, and further increased after 7 days.^75^, ^88^ The increased MCUb expression caused decreased mitochondrial Ca^2+^ uptake and suppressed mPTP opening, thus reducing cardiac tissue damage.^88^ Due to the absence of MCUb until 3 days post‐I/R, MCUb may play a role in limiting infarct expansion and post‐ischemic pathological remodeling, which occurs days following initial injury.^75^, ^88^

#### 3.4.1 Cardiac arrhythmia

Arrhythmia is a common cause of cardiovascular disease‐related deaths, and there are limited treatment options due to adverse anti‐arrhythmic drug side effects.^89^ Cardiac arrhythmia is associated with cellular Ca^2+^ dysregulation in cardiomyocytes.^89^ Mitochondria absorb ryanodine receptor 2 (RyR2)‐mediated Ca^2+^ released from the sarcoplasmic reticulum (SR).^89^ RyR2 channel clusters are in close proximity to the mitochondria.^90^ Thus, enhancing the ability of mitochondria‐mediated Ca^2+^ uptake can interfere with Ca^2+^ diffusion between RyR2 clusters, reducing the probability of arrhythmogenic spontaneous Ca^2+^ wave initiation and propagation.^90^ Intracellular Ca^2+^ transporters, such as mtCU, may be candidate pathways for novel anti‐arrhythmic therapeutics with fewer adverse side effects.^89^

Diabetes mellitus increases the risk of heart failure due to decreased cardiomyocyte function, linked to changes in cardiac mitochondrial energy metabolism.^91^ Diabetes mellitus is a remarkable proponent of cardiac arrhythmias.^92^, ^93^ Type 1 diabetic mice hearts, induced using STZ, were found to have altered expression of MCU and MCUb, resulting in a decrease in mitochondrial Ca^2+^ uptake and cardiac function.^91^ Reduced rates of contraction and relaxation were observed in isolated‐perfused diabetic hearts.^91^ MCU and EMRE protein levels decreased by 50% and 36%, respectively, in the mouse hearts 8 weeks post‐STZ, and MCUb protein levels were increased by 31% compared to control hearts.^91^ Additionally, in type 2 diabetic mouse hearts, MCUb was found to be upregulated.^94^ Gene therapy displacement of endogenous *MCUB* with a dominant‐negative *MCUB* transgene (*MCUB* ^W245R/V251E^) in vivo rescued cardiomyocytes from relying almost solely on mitochondrial fatty acids for energy production and increased cardiac contractile function.^94^ Interestingly, the MCUb^W245R/V251E^ mutant was found to interact with MICU1, but similar to the proteomic data (see Section 3.1) WT MCUb did not interact with MICU1 in cardiomyocytes from the type 2 diabetic mouse hearts.^94^ Further, the MCUb^W245R/V251E^ mutant increased mitochondrial Ca^2+^ uptake from the reduced rates observed with overexpression of FLAG‐tagged MCUb, to rates similar to cells expressing FLAG‐tagged MCU.^94^ Thus, MCUb remains at a pivotal interface between metabolism and cardiac function, with the reduction of MCUb inhibitory function holding considerable promise for the chronically stressed heart.^94^

Nonischemic heart failure (HF) triggered by Ca^2+^ dysregulation also contributes to arrhythmia risk.^95^ Isolated myopathic myocytes in nonischemic HF mice showed increased mitochondrial Ca^2+^ transients and action potential duration, which both decreased upon knockdown of *MCU*.^95^ This knockdown also reduced ventricular fibrillation in these nonischemic HF mice.^95^ Interestingly, human cardiac samples isolated from HF patients showed an ~2‐fold increase in the MICU1/MCU protein ratio compared to non‐HF control samples.^96^ Yet, there was no change in either MCU or MCUb protein levels in the human HF cardiac samples compared to the non‐HF control samples.^96^ These data contrast the MCUb upregulation observed in I/R injury mouse models (see Section 3.4; Ref. [66, 88]). While differences in beat frequency/energetic demands of the mouse and human heart, could underlie some inconsistencies observed in mtCU subunit contribution to pathogenesis, it is noteworthy that MCUb expression changes were only observed ~2–3 days post‐I/R injury in mice.^66^, ^88^ Thus, a careful analysis of the temporal expression pattern of MCUb in human HF and mouse HF models is needed for comparison.

## 4 CONSERVATION OF MCU‐IDENTIFIED POST‐TRANSLATIONAL MODIFICATION SITES IN MCUb

**FIGURE 5:**
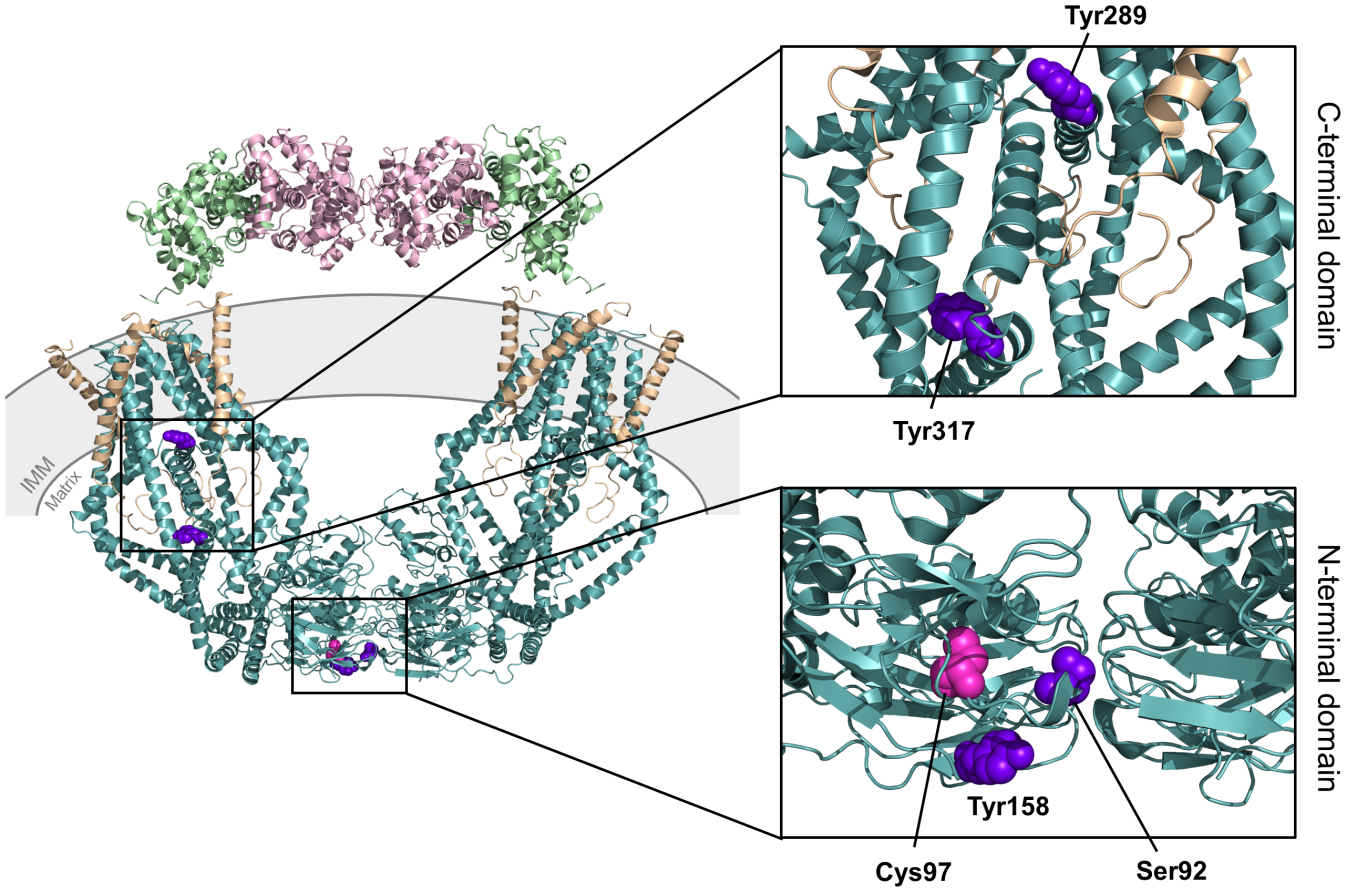
Three‐dimensional structures of the human mitochondrial calcium uniporter (MCU) holocomplex elucidated in a high Ca^2+^/non‐occluded state, highlighting the known post‐translational modification residue positions. The structure images were generated using the 6WDO.pdb.^31^ Note that the Ser57 phosphorylation site is not visible in this structure. The MCU complex includes MCU (teal), EMRE (beige), MICU1 (green), and MICU2 (pink). The righthand panels show zoomed views of the post‐translational modification sites occurring in the C‐terminal domain (top) and the N‐terminal domain (bottom). The known phosphorylation residue positions are shown with purple spheres, and the *S‐*glutathionylation position is shown with magenta spheres. EMRE, essential MCU regulatory protein, MICU1/2, mitochondrial Ca^2+^ uptake 1 and −2 proteins; IMM, inner mitochondrial membrane.

Post‐translational modifications regulate mtCU channel oligomerization and function,^40^ but the effects on the MCU paralog, MCUb, have yet to be characterized and understood. For instance, proline‐rich tyrosine kinase 2 (Pyk2)‐dependent phosphorylation of Tyr158 in the N‐terminal domain and Tyr289/Tyr317 in the C‐terminal domain of MCU was determined to increase mitochondrial Ca^2+^ uptake and mitochondrial reactive oxygen species (mROS) production, triggering mPTP opening and cell death (Table 2; Figure 5; Ref. [22, 40, 99]). Phosphorylation of Ser57 within the MCU N‐terminal domain promoted mitochondrial Ca^2+^ uptake, necessary for proper mitotic progression (Table 2; Ref. [22, 98]). Ser57 of MCU contains an AMP‐activated protein kinase (AMPK) consensus phosphorylation site, also indicated as a Ca^2+^/calmodulin‐dependent protein kinase II (CAMKII) phosphorylation site.^97^, ^98^ Phosphorylation of Ser92 within the MCU N‐terminal domain has been shown to disrupt the Ser92:Asp119 hydrogen bond between loop2 and loop4 of the β‐grasp‐fold (Table 2; Figure 5; Ref. [15]). The resultant conformational changes, due to the loss of the hydrogen bond and increased negative charge, were found to disrupt the dimerization between mtCU channel tetramers.^15^ However, whether phosphorylation regulates MCUb inhibitory action remains elusive.

**TABLE 2:** Summary of the post‐translational modifications known to regulate MCU and conservation in MCUb

Post‐translational modification	Residue position in MCU	Domain position of residue	Conservation in MCUb	References
Phosphorylation	Ser57	N‐terminal domain	No (Asn42)	Joiner et al., (2012)^97^; Zhao et al., (2019)^98^; Alevriadou et al., (2021)^22^
Phosphorylation	Ser92	N‐terminal domain	Yes (Ser77)	Joiner et al., (2012)^97^; Nemani et al., (2018)^2^; Lee et al., (2020)^15^
Phosphorylation	Tyr158	N‐terminal domain	Yes (Tyr143)	Alevriadou et al., (2021)^22^; Feno et al., (2021)^40^
Phosphorylation	Tyr289	C‐terminal domain	Yes (Tyr274)	O‐Uchi et al., (2014)^99^; Feno et al., (2021)^40^
Phosphorylation	Tyr317	C‐terminal domain	Yes (Tyr302)	Feno et al., (2021)^40^
*S*‐glutathionylation	Cys97	N‐terminal domain	Yes (Cys82)	Dong et al., (2017)^100^; Nemani et al., (2018)^2^

MCU also contains three Cys in the N‐terminal domain (i.e., Cys67, Cys97, Cys191) that may be susceptible to oxidative modifications.^2^, ^100^ Indeed, *S*‐glutathionylation of Cys97 within the MCU N‐terminal domain has been shown to regulate mtCU channel activity (Table 2; Figure 5; Ref. [100]). Oxidation and mutation of Cys97 in MCU both induce a conformational change within the N‐terminal β‐grasp fold, resulting in elevated mitochondrial Ca^2+^ uptake through the mtCU channel, increased mROS, and an increased propensity for mitochondrial Ca^2+^ overload‐induced cell death.^100^ While a biochemical gel shift assay revealed that MCU is the luminal mROS sensor,^2^, ^100^ the Cys97 is conserved in MCUb (i.e., existing as Cys82; Figure 1) and may also play a regulatory role under conditions that favor MCUb expression and oxidative modifications. Interestingly, mutating Cys97 of MCU abrogates the inhibitory action of Ru265 (see Section 3.2), suggesting that this post‐translational modification site may also play a role in binding this small molecule inhibitor.^35^

The high sequence homology with MCU implies that post‐translational modifications of MCUb could also contribute to the regulation of MCUb and mtCUs in which the dominant‐negative beta subunit has been incorporated. Thus, understanding the mechanisms and structural consequences of post‐translational modifications within MCUb will provide a deeper understanding of the subunit regulation and function, both within physiological and pathophysiological states.

## 5 CONCLUSION

Since the discovery of mtCU components, MCUb has emerged as a critical regulator, despite the lack of understanding surrounding the precise molecular mechanisms that govern the complex assembly and negative regulation by this inhibitor.^30^ Determining the precise structural mechanisms that underlie the inhibitory and differential function of MCUb compared to MCU requires considerable future efforts. Nevertheless, this type of research focus could be critical for the development of novel research tools and pave the way to new therapeutics. The research focused on the less well‐studied regulatory components of mtCU will also aid in the understanding of numerous pervasive pathologies effected by Ca^2+^ overload.^30^ For example, with cardiac arrhythmias being a global health burden impacting close to 2% of the population and only moderately effective treatment options available,^101^ research focusing on the association with mitochondrial Ca^2+^ regulation and the MCUb subunit will lead to a greater understanding of arrhythmogenesis and help in the development of safer therapeutics.

## AUTHOR CONTRIBUTIONS

Danielle M. Colussi: writing original draft, investigation, editing/revising, conceptualization, and model analyses. Peter B. Stathopulos: editing, review, supervision, modeling methodology, and funding. Both authors read and approved the submitted version

## DISCLOSURES

The authors declare that the research was conducted in the absence of any commercial or financial relationships that could be construed as a potential conflict of interest.

## Supporting information


Supplementary Material S1

Supplementary Material S2

## Data Availability

The data that support the findings of this study are available in the supplementary materials of this article as Supplementary Dataset 1 (PISA and PyMOL analysis spreadsheet), Supplementary Dataset 2 (MCUb homotetramer homology model coordinates—non‐occluded), Supplementary Dataset 3 (MCU‐MCUb heterotetramer homology model coordinates—non‐occluded), Supplementary Dataset 4 (MCUb homotetramer homology model coordinates—occluded), Supplementary Dataset 5 (MCU‐MCUb heterotetramer homology model coordinates—occluded). The MCU homotetramer coordinates are openly available in the Protein Data Bank at https://www.rcsb.org/, reference 6WDO.pdb and 6WDN.pdb. The human MCU and MCUb protein sequences were taken from https://www.ncbi.nlm.nih.gov/protein/, references NP_612366.1 and NP_060388.2, respectively.
